# Associação entre desrespeito e abuso durante o parto e o risco de depressão pós-parto: estudo transversal

**DOI:** 10.1590/0102-311XPT008024

**Published:** 2024-09-16

**Authors:** Haylane Nunes da Conceição, Alberto Pereira Madeiro

**Affiliations:** 1 Programa de Pós-graduação em Saúde e Comunidade, Universidade Federal do Piauí, Teresina, Brasil.

**Keywords:** Violência Obstétrica, Depressão Pós-parto, Parto, Violência Contra a Mulher, Saúde Materna, Obstetric Violence, Postpartum Depression, Parturition, Violence Against Women, Maternal Health, Violencia Obstétrica, Depresión Posparto, Parto, Violencia Contra la Mujer, Salud Materna

## Abstract

O objetivo deste estudo é analisar a relação entre desrespeito e abuso durante o parto e o risco de depressão pós-parto. Trata-se de estudo transversal, realizado com mulheres das zonas rural e urbana de Caxias, Maranhão, Brasil. Considerou-se a depressão pós-parto como variável dependente, avaliada pela *Escala de Depressão Pós-Natal de Edimburgo*. As variáveis independentes foram características sociodemográficas, antecedentes de saúde mental, aspectos comportamentais, características obstétricas e autopercepção do desrespeito e abuso durante o parto. Empregou-se o teste do qui-quadrado de Pearson e a regressão logística múltipla para avaliar a associação entre depressão pós-parto e desrespeito e abuso durante o parto. Foram entrevistadas 190 mulheres. A depressão pós-parto apresentou prevalência de 16,3%. A ocorrência de pelo menos um tipo de desrespeito e abuso durante o parto foi de 97,4%, com predomínio das condições do sistema de saúde e restrições (94,7%). Mais da metade das mulheres (66,3%) foram submetidas a dois tipos de desrespeito e abuso durante o parto, enquanto três ou mais formas foram relatadas por 22,6%. Sofrer duas (OR_ajustada_ = 3,01; IC95%: 1,08-8,33) e três ou mais formas de desrespeito e abuso durante o parto (OR_ajustada_ = 3,41; IC95%: 1,68-24,40) aumentou a chance da ocorrência de depressão pós-parto. Houve associação significativa entre desrespeito e abuso durante o parto e depressão pós-parto, e o atendimento digno e respeitoso às mulheres durante o parto pode reduzir os riscos da sintomatologia de depressão pós-parto.

## Abstract (en)

This study aimed to analyze the relationship between disrespect and abuse during labor and the risk of postpartum depression. This is a cross-sectional study carried out with women from the rural and urban areas of Caxias, Maranhão State, Brazil. Postpartum depression was considered the dependent variable, assessed using the *Edinburgh Postnatal Depression Scale*. The independent variables were sociodemographic characteristics, mental health history, behavioral aspects, obstetric characteristics and self-perception of disrespect and abuse during labor. Pearson’s chi-square test and multiple logistic regression were used to assess the association between postpartum depression and disrespect and abuse during labor. A total of 190 women were interviewed. The prevalence of postpartum depression was 16.3%. The occurrence of at least one type of disrespect and abuse during labor was 97.4%, with health system conditions and restrictions predominating (94.7%). More than half of the women (66.3%) suffered two forms of disrespect and abuse during labor, while three or more forms were reported by 22.6%. Suffering two (_adjusted_OR = 3.01; 95%CI 1.08-8.33) and three or more forms of disrespect and abuse during labor (_adjusted_OR = 3.41; 95%CI: 1.68-24.40) increased the chance of postpartum depression. There was a significant association between disrespect and abuse during labor and postpartum depression, and dignified and respectful care for women during childbirth were found to reduce the risk of postpartum depression symptoms.

## Abstract (es)

El objetivo de este estudio fue analizar la relación entre la falta de respeto y el abuso durante el parto y el riesgo de depresión posparto. Se trata de un estudio transversal, realizado con mujeres de la zona rural y urbana de Caxias, Maranhão, Brasil. La depresión posparto fue considerada como una variable dependiente, evaluada por la *Escala de Depresión Posnatal de Edimburgo*. Las variables independientes fueron características sociodemográficas, antecedentes de salud mental, aspectos comportamentales, características obstétricas y autopercepción de falta de respeto y el abuso durante el parto. Se empleó la prueba de chi-cuadrado de Pearson y la regresión logística múltiple para evaluar la asociación entre depresión posparto y falta de respeto y el abuso durante el parto. Se entrevistó a 190 mujeres. La depresión posparto tuvo una prevalencia del 16,3%. La ocurrencia de al menos un tipo de falta de respeto y el abuso durante el parto fue del 97,4%, con predominio de las condiciones del sistema de salud y restricciones (94,7%). Más de la mitad de las mujeres (66,3%) padecieron dos tipos de falta de respeto y el abuso durante el parto, mientras que tres o más formas fueron referidas por el 22,6%. Sufrir dos (OR_ajustado_ = 3,01; IC95%: 1,08-8,33) y tres o más formas de falta de respeto y el abuso durante el parto (OR_ajustado_ = 3,41; IC95%: 1,68-24,40) aumentó la posibilidad de que se produjera depresión posparto. Hubo una asociación significativa entre falta de respeto y el abuso durante el parto y depresión posparto, y una atención digna y respetuosa a las mujeres durante el parto puede reducir los riesgos de los síntomas de depresión posparto.

## Introdução

A depressão é a principal causa de mortes por suicídio em todo o mundo ^1^. No pós-parto, a ocorrência de depressão, denominada de depressão pós-parto, configura-se como transtorno depressivo de gravidade moderada a grave que pode ocorrer até o primeiro ano ^2^ ^,^ ^3^. Caracteriza-se por sentimento de tristeza, inutilidade ou culpa, fadiga, distúrbios do sono, baixa concentração, perda de apetite e interesse em atividades habituais, pensamentos de morte, ideações suicidas e cuidados excessivos ou inapropriados com o filho ^1^ ^,^ ^3^.

A depressão pós-parto afeta aproximadamente 17% das mulheres globalmente ^4^. No Brasil, uma pesquisa de âmbito nacional de 2011-2012 com 23.894 puérperas, evidenciou prevalência de 26% ^5^. Contudo, outros estudos nacionais demonstraram taxas variando de 5,7% ^6^ a 19,8% ^7^. A ocorrência de depressão pós-parto pode ocasionar inúmeras repercussões negativas, tais como maior probabilidade de problemas emocionais, cognitivos e comportamentais na criança, descontinuidade precoce da amamentação e ideação suicida ^8^. Entre os principais fatores associados à depressão pós-parto estão níveis socioeconômicos mais baixos, histórico de depressão e pequeno apoio social e conjugal ^3^. Além desses, desrespeito e abuso durante o parto vêm sendo apontados como relevantes preditores do transtorno ^9^ ^,^ ^10^.

O desrespeito e abuso durante o parto, também conhecido pelos termos maus-tratos no parto e violência obstétrica ^11^, pode ser manifestado como abuso físico, abuso verbal, abuso sexual, discriminação, não cumprimento de normas profissionais recomendadas e relações inadequadas entre mulheres e profissionais de saúde ^12^. Sabe-se que esse evento resulta frequentemente da interação entre os profissionais de saúde e a mulher, porém as condições e restrições dos sistemas de saúde também influenciam sua ocorrência, com destaque para as más condições de trabalho, que incluem falta de equipamento, de medicamentos e de profissionais ^12^ ^,^ ^13^.

Mulheres submetidas a desrespeito e abuso durante o parto apresentam probabilidade mais elevada de manifestarem depressão pós-parto ^9^. Um estudo realizado em Gana, Guiné, Myanmar e Nigéria, entre 2016 e 2017, observou que o abuso físico ou verbal no parto aumenta em aproximadamente duas vezes a chance de depressão pós-parto moderada a grave ^10^. No Brasil, investigações no Distrito Federal ^14^ e em Pelotas, Rio Grande do Sul ^6^, também identificaram que as mulheres expostas a desrespeito e abuso durante o parto apresentam maior risco de ocorrência de sintomas de depressão pós-parto.

Apesar desses dados, ainda se conhece pouco sobre a relação entre desrespeito e abuso durante o parto e a ocorrência de depressão pós-parto, sendo que a escassez de estudos sobre as repercussões do desrespeito e abuso durante o parto é uma das principais lacunas sobre o tema na atualidade ^9^ ^,^ ^12^. Nenhuma pesquisa, por exemplo, investigou a associação entre desrespeito e abuso durante o parto e depressão pós-parto na Região Nordeste do Brasil, onde o modelo de atenção ao parto ainda apresenta práticas intervencionistas e de maus-tratos ^15^. Desse modo, o objetivo deste estudo é analisar a relação entre desrespeito e abuso durante o parto e o risco de depressão pós-parto.

## Métodos

Trata-se de estudo transversal, realizado com mulheres residentes nas zonas rural e urbana de Caxias, Maranhão, Brasil, que receberam atendimento durante o trabalho de parto e parto na maternidade do município no período de dezembro de 2022 a junho de 2023. Foram excluídas aquelas cujos recém-nascidos foram natimortos, ou que apresentassem surdez ou diminuição da acuidade auditiva, deficiência cognitiva ou problemas de linguagem.

O tamanho amostral foi determinado utilizando o cálculo para amostras probabilísticas finitas, levando-se em consideração o número de nascidos vivos em Caxias no ano de 2020 (n = 3.165) ^16^, prevalência de depressão pós-parto de 26% nas mulheres brasileiras ^5^, nível de 95% de confiança, erro de 5% e acréscimo de 10% para perdas.

A coleta de dados ocorreu em duas etapas. Inicialmente, foram obtidas informações cadastrais (nome, endereço e data do parto) das mulheres cujos partos ocorreram entre os meses de dezembro de 2022 e junho de 2023. Na segunda etapa, com o auxílio dos agentes comunitários de saúde, foram realizadas entrevistas no domicílio das mulheres três meses após o parto (março a setembro de 2023). Antes do início da coleta de dados, um teste piloto com dez mulheres no terceiro mês de pós-parto foi conduzido com o intuito de aprimorar as perguntas.

Os dados foram obtidos por meio de entrevista estruturada, face a face, utilizando três instrumentos. O primeiro continha questionário com: (1) variáveis sociodemográficas (faixa etária; raça/cor da pele; escolaridade; parceria conjugal; ocupação profissional; renda familiar); (2) antecedente de saúde mental da mulher (transtorno de depressão diagnosticado pelo médico); (3) aspectos comportamentais relacionados à saúde no período gestacional (consumo de cigarro; consumo de álcool; consumo de drogas ilícitas); e (4) características obstétricas (histórico de aborto; número de partos; se a gestação foi planejada; sentimento em relação à gestação [caso não planejada]; número de consultas do pré-natal; idade gestacional no momento do parto; tipo de parto; presença de acompanhante no trabalho de parto e no parto).

O segundo instrumento foi a *Escala de Depressão Pós-Natal de Edimburgo* (EPDS), que investigou os sintomas depressivos após o parto. A EPDS é um instrumento composto por dez itens. Cada item pode pontuar de 0 a 3, com escore que varia de 0 a 30, sendo que as pontuações mais altas indicam maior risco de quadro depressivo ^17^. Neste estudo, considerou-se como risco de depressão pós-parto pontuação de corte > 13. Essa pontuação foi previamente validada no Brasil, apresentando sensibilidade 59,5% e especificidade de 88,4% no diagnóstico de depressão pós-parto ^18^. Por fim, o terceiro instrumento avaliou a autopercepção de desrespeito e abuso durante o trabalho de parto e/ou o parto.

O desrespeito e abuso durante o parto foi avaliado por meio de sete dimensões: (1) abuso físico (“Você foi espancada, esbofeteada, chutada ou beliscada durante o parto?”; “Você foi contida na cama ou amordaçada durante o parto?”); (2) abuso verbal (“Utilizaram uma linguagem dura ou rude?”; “Fizeram comentários condenando ou acusando você de algo?”; “Você recebeu ameaças de tratamento inadequado ou foi culpada por resultados ruins no parto?”); (3) abuso sexual (“Você recebeu algum comentário sexualmente degradante?”; “Houve algum contato ou toque físico sexual indesejado, incluindo penetração oral, vaginal ou oral?”); (4) discriminação (“Você foi discriminada por causa da sua raça?” “Religião?”; “Idade?”; “Condição econômica?”); (5) não cumprimento de normas profissionais recomendadas (“Os profissionais se comunicaram muito pouco com você?”; “Os profissionais usaram excessivamente a linguagem técnica?”; “Você não recebeu apoio por parte dos profissionais de saúde?”; “Você não podia se movimentar?”; “Não tiveram respeito pela sua posição de parto preferida?”); (6) relações inadequadas entre mulheres e profissionais de saúde (“Fizeram algo sem o seu consentimento?”; “Compartilharam alguma informação confidencial sobre você?”; “Fizeram exames vaginais dolorosos?”; “Houve recusa em aliviar sua dor?”; “Fizeram alguma cirurgia sem sua permissão?”; “Você foi abandonada por longos períodos?”); e (7) condições do sistema de saúde e restrições (“A maternidade tem infraestrutura física inadequada?”; “Os profissionais de saúde e funcionários da maternidade são poucos ou estão ausentes?”; “Na maternidade há falta de privacidade?”; “Na maternidade faltam leitos?”) ^12^. Considerou-se desrespeito e abuso durante o parto como a resposta positiva a pelo menos um dos itens das dimensões.

Os dados foram descritos por frequências absolutas e relativas no programa SPSS versão 20.0 (https://www.ibm.com/). Empregou-se o teste do qui-quadrado de Pearson para avaliar a associação entre o desfecho (depressão pós-parto) e a ocorrência de desrespeito e abuso durante o parto, calculando-se *odds ratio* bruta (OR_bruta_) e intervalos de 95% de confiança (IC95%). Foi também avaliada a associação de depressão pós-parto com um, dois e três ou mais tipos de desrespeito e abuso durante o parto, assim como a associação de depressão pós-parto com cada uma das dimensões de desrespeito e abuso durante o parto.

Realizou-se análise multivariada, por meio de regressão logística múltipla, com cálculo de OR ajustada (OR_ajustada_) e IC95%, cujo modelo foi ajustado pelas variáveis faixa etária, raça/cor da pele, escolaridade, transtorno de depressão anterior e gestação planejada, tendo como referência estudos prévios ^6^ ^,^ ^14^ ^,^ ^19^. Todas as variáveis que mostraram valor de p < 0,20 foram incluídas na análise multivariada. O nível de significância considerado foi de 5%.

A pesquisa foi aprovada pelo Comitê de Ética em Pesquisa da Universidade Federal do Piauí (CAAE 64878022.9.0000.5214, parecer nº 5.878.965).

## Resultados

A maioria das 190 mulheres entrevistadas tinha entre 20-29 anos (51,6%), raça/cor da pele parda (66,3%), tinha cursado o Ensino Médio (62,1%), nenhuma ocupação profissional (64,2%) e renda familiar entre 1-2 salários mínimos (56,3%). Observou-se, também, predomínio de mulheres com parceria conjugal (69,5%) e sem histórico de depressão (94,2%). O consumo de cigarro (5,3%) e álcool (14,7%) durante o período gestacional foi relatado por pequeno percentual de participantes. O risco de depressão pós-parto foi mais frequente entre aquelas com 40-49 anos (22,2%), raça/cor da pele amarela (20%), Ensino Médio completo (19,5%), parceria conjugal (16,7%), sem ocupação profissional (18%) e renda inferior a 1 salário mínimo (22,1%), com histórico de depressão (54,5%), sem consumo de cigarro (17,2%) e com consumo de álcool (17,9%) durante a gestação (Tabela 1).

**Tabela 1:** Características sociodemográficas, antecedentes de saúde mental, aspectos comportamentais relacionados à saúde e a ocorrência de depressão pós-parto. Caxias, Maranhão, Brasil, 2023.

Variáveis	Total (N = 190)	Depressão pós-parto	
		Sim	Não
	n (%)	n (%)	n (%)
Faixa etária (anos)			
10-19	31 (16,3)	1 (3,2)	30 (96,8)
20-29	98 (51,6)	17 (17,3)	81 (82,7)
30-39	52 (27,4)	11 (21,2)	41 (78,8)
40-49	9 (4,7)	2 (22,2)	7 (77,8)
Raça/Cor da pele			
Branca	12 (6,3)	1 (8,3)	11 (91,7)
Preta	47 (24,7)	7 (14,9)	40 (85,1)
Parda	126 (66,3)	22 (17,5)	104 (82,5)
Amarela	5 (2,6)	1 (20,0)	4 (80,0)
Escolaridade			
Ensino Fundamental	42 (22,1)	6 (14,3)	36 (85,7)
Ensino Médio	118 (62,1)	23 (19,5)	95 (80,5)
Ensino Superior	30 (15,8)	2 (6,7)	28 (93,3)
Parceria conjugal			
Não	58 (30,5)	9 (15,5)	49 (84,5)
Sim	132 (69,5)	22 (16,7)	110 (83,3)
Ocupação profissional			
Não	122 (64,2)	22 (18,0)	100 (82,0)
Sim	68 (35,8)	9 (13,2)	59 (86,8)
Renda familiar (salários mínimos)			
< 1	68 (35,8)	15 (22,1)	53 (77,9)
1-2	107 (56,3)	15 (14,0)	92 (86,0)
3-5	15 (7,9)	1 (6,7)	14 (93,3)
Transtorno de depressão			
Não	179 (94,2)	25 (14,0)	154 (86,0)
Sim	11 (5,8)	6 (54,5)	5 (45,5)
Uso de cigarro			
Não	180 (94,7)	31 (17,2)	149 (82,8)
Sim	10 (5,3)	0 (0,0)	10 (100,0)
Uso de álcool			
Não	162 (85,3)	26 (16,0)	136 (84,0)
Sim	28 (14,7)	5 (17,9)	23 (82,1)
Uso de drogas ilícitas			
Não	190 (100,0)	31 (16,3)	159 (83,7)

A Tabela 2 apresenta as características obstétricas das mulheres. Houve maior frequência de mulheres sem histórico de aborto (75,8%), com 2-4 partos (53,2%) e com planejamento da última gestação (52,1%). Entre aquelas cujas gestações não foram planejadas (47,9%), a maioria foi desejada (88%). Além disso, a maior parte realizou mais de 6 consultas pré-natais (84,2%), tinha idade gestacional entre 37-42 semanas (88,4%) e evoluiu com parto vaginal (51,1%). Chama a atenção que pouco menos da metade (49,5%) teve acompanhante durante o trabalho de parto e/ou o parto. As mulheres identificadas com risco de depressão pós-parto tinham percentual mais elevado de histórico de aborto (17,4%), tinham mais de 4 partos (38,5%), não haviam planejado a última gestação (16,5%), que era indesejada (40%), tiveram 6 ou menos consultas pré-natais (30%), idade gestacional menor que 37 semanas (27,8%), partos vaginais (17,5%) e sem acompanhante (17,7%).

O risco de depressão pós-parto foi identificado em 16,3% das participantes. Por sua vez, a experiência de pelo menos 1 tipo de desrespeito e abuso durante o parto foi relatado por quase todas as mulheres (97,4%). Mais da metade delas (66,3%) foram submetidas a 2 tipos de desrespeito e abuso durante o parto e os relatos de 3 ou mais formas de atendimento desrespeitoso e abusivo foram identificados em 22,6% das mulheres (Tabela 3).

**Tabela 2:** Características obstétricas e a ocorrência de depressão pós-parto. Caxias, Maranhão, Brasil, 2023.

Variáveis	Total (N = 190)	Depressão pós-parto	
		Sim	Não
	n (%)	n (%)	n (%)
Histórico de aborto			
Não	144 (75,8)	23 (16,0)	121 (84,0)
Sim	46 (24,2)	8 (17,4)	38 (82,6)
Número de partos			
1	76 (40,0)	8 (10,5)	68 (89,5)
2-4	101 (53,2)	18 (17,8)	83 (82,2)
> 4	13 (6,8)	5 (38,5)	8 (61,5)
Gestação planejada			
Não	91 (47,9)	15 (16,5)	76 (83,5)
Sim	99 (52,1)	16 (16,2)	83 (83,8)
Sentimento em relação à gestação não planejada (n = 90)			
Desejada	80 (88,9)	11 (13,7)	69 (86,3)
Não desejada	10 (11,1)	4 (40,0)	6 (60,0)
Número de consultas pré-natais			
6 ou menos	30 (15,8)	9 (30,0)	21 (70,0)
Mais de 6	160 (84,2)	22( 13,8)	138 (86,3)
Idade gestacional (semanas)			
< 37	18 (9,5)	5 (27,8)	13 (72,2)
37-42	168 (88,4)	25 (14,9)	143( 85,1)
> 42	4 (2,1)	1 (25,0)	3 (75,0)
Tipo de parto			
Vaginal	97 (51,1)	17 (17,5)	80 (82,5)
Cesárea	93 (48,9)	14 (15,0)	79 (85,0)
Acompanhante durante parto			
Não	96 (50,5)	17 (17,7)	79 (82,3)
Sim	94 (49,5)	14 (14,9)	80 (85,1)

**Tabela 3:** Depressão pós-parto e desrespeito e abuso no parto. Caxias, Maranhão, Brasil, 2023.

Variáveis	n (%)
Depressão pós-parto	
Não	159 (83,7)
Sim	31 (16,3)
Desrespeito e abuso durante o parto (1 categoria)	
Não	5 (2,6)
Sim	185 (97,4)
Desrespeito e abuso durante o parto (2 categorias)	
Não	64 (33,7)
Sim	126 (66,3)
Desrespeito e abuso durante o parto (3 ou mais categorias)	
Não	147 (77,4)
Sim	43 (22,6)

Houve relato mais frequente de desrespeito e abuso durante o parto relacionado às condições do sistema de saúde e restrições (94,7%), seguida por relações inadequadas entre mulheres e profissionais de saúde (63,7%), não cumprimento de normas profissionais recomendadas (17,9%), abuso verbal (16,3%), abuso físico (2,1%) e discriminação (1,1%). As principais condições do sistema de saúde e restrições relatadas foram falta de recursos (21,7%) e falta de recursos/política (72,2%). Entre os tipos de relações inadequadas houve destaque para falta de apoio (50,4%) e falta de apoio e perda de autonomia (21,5%). Negligência e abandono (20,6%) e falta de consentimento informado/confidencialidade (26,5%) foram os tipos mais mencionados de não cumprimento das normas profissionais recomendadas. Os abusos verbais ocorreram por meio de linguagem dura (87,1%) e linguagem dura/ameaças/culpabilização (9,7%). Já o abuso físico foi manifestado pela utilização da força física (100%). A discriminação relatada foi em decorrência da raça/religião (50%) e raça/religião/idade (50%) (Tabela 4).

**Tabela 4:** Tipos de desrespeito e abuso durante o parto. Caxias, Maranhão, Brasil, 2023.

Variáveis	n (%) *^,^**
Abuso físico	4 (2,1)
Força física	4 (100,0)
Abuso verbal	31 (16,3)
Linguagem dura	27 (87,1)
Linguagem dura/Ameaças/Culpabilização	3 (9,7)
Ameaças/Culpabilização	1 (3,2)
Discriminação	2 (1,1)
Raça/Religião	1 (50,0)
Raça/Religião/Idade	1 (50,5)
Não cumprimento de normas profissionais recomendadas	34 (17,9)
Falta de consentimento informado/confidencialidade	9 (26,5)
Negligência/Abandono	7 (20,6)
Exame físico/Procedimentos	5 (14,7)
Falta de consentimento informado/confidencialidade/exame físico/procedimentos	3 (8,8)
Falta de consentimento informado/negligência/abandono	5 (14,7)
Exame físico/Procedimentos/Negligência/Abandono	5 (14,7)
Relações inadequadas de profissionais de saúde	121 (63,7)
Falta de apoio	61 (50,4)
Falta de apoio e perda de autonomia	26 (21,5)
Perda da autonomia	25 (20,7)
Comunicação ineficaz/não efetiva	5 (4,1)
Comunicação ineficaz/não efetiva/falta de apoio/perda de autonomia	2 (1,7)
Comunicação ineficaz/não efetiva/falta de apoio	1 (0,8)
Comunicação ineficaz/não efetiva/perda de autonomia	1 (0,8)
Condições do sistema de saúde e restrições	180 (94,7)
Falta de recursos/Falta de políticas	130 (72,2)
Falta de recursos	39 (21,7)
Falta de políticas	11 (6,1)

* Pode ocorrer relato de mais de uma dimensão de desrespeito e abuso durante o parto;

** Os percentuais dos subtipos foram calculados em relação ao total da dimensão em que eles estão inseridos.

A análise bivariada demonstrou associação estatisticamente significativa entre depressão pós-parto e o relato de pelo menos 1 tipo de desrespeito e abuso durante o parto (OR_bruta_ = 0,12; IC95%: 0,02-0,74). Sofrer 2 formas de desrespeito e abuso durante o parto (OR_bruta_ = 3,07; IC95%: 1,12-8,42), 3 ou mais tipos de desrespeito e abuso durante o parto (OR_bruta_ = 1,82; IC95%: 1,78-4,23) e abuso verbal (OR_bruta_ = 3,13; IC95%: 1,30-7,56) também aumentaram a chance da ocorrência de depressão pós-parto. Na análise multivariada, as mulheres que foram expostas a 2 (OR_ajustada_ = 3,01; IC95%: 1,08-8,33) e 3 ou mais formas de desrespeito e abuso durante o parto (OR_ajustada_ = 3,41; IC95%: 1,68-24,40) apresentaram maior chance de desenvolverem depressão pós-parto, quando comparadas àquelas que não sofreram tratamento desrespeitoso e abusivo durante o parto (Tabela 5).

**Tabela 5:** Associação de desrespeito e abuso no parto a ocorrência de depressão pós-parto. Caxias, Maranhão, Brasil, 2023.

Variáveis	Depressão pós-parto					
	Não ajustado			Ajustado *		
	OR_bruta_	IC95%	Valor de p	OR_ajustada_	IC95%	Valor de p
Desrespeito e abuso durante o parto (1 categoria)	0,12	0,02-0,74	0,007	0,23	0,02-2,28	0,101
Desrespeito e abuso durante o parto (2 categorias)	3,07	1,12-8,42	0,024	3,01	1,08-8,33	0,035 **
Desrespeito e abuso durante o parto (3 ou mais categorias)	1,82	1,78-4,23	0,041	3,41	1,68-24,40	0,011 **
Abuso verbal	3,13	1,30-7,56	0,009	2,19	0,84-5,68	0,109
Discriminação	5,27	0,32-86,54	0,300	-	-	-
Não cumprimento	1,78	0,72-4,41	0,209	-	-	-
Relações inadequadas	2,19	0,89-5,29	0,082	1,86	0,73-4,32	0,191
Condições do sistema	0,43	0,10-1,76	0,229	-	-	-

OR: *odds ratio*; IC95%: intervalos de 95% de confiança.

* Modelo ajustado por idade, raça/cor da pele, escolaridade, renda familiar, histórico de depressão, gestação planejada;

** Valor de p < 0,05.

## Discussão

Este estudo é o primeiro a investigar a relação entre desrespeito e abuso durante o parto e depressão pós-parto em um município da Região Nordeste do Brasil. Os resultados evidenciaram que menos de um quarto das entrevistadas apresentou risco de depressão pós-parto. Por outro lado, quase a totalidade sofreu alguma experiência de desrespeito e abuso durante o parto, enquanto mais da metade foi submetida simultaneamente a 2 tipos de desrespeito e abuso durante o parto e cerca de um quarto delas a 3 ou mais formas. As condições do sistema de saúde e restrições, especialmente a falta de recursos e políticas, seguido por relações inadequadas entre mulheres e profissionais de saúde e o não cumprimento de normas profissionais recomendadas, foram os tipos mais relatados. Os dados demonstraram também que as mulheres expostas a 2 e 3 ou mais formas de desrespeito e abuso durante o parto tiveram aproximadamente três vezes mais chances de desenvolverem depressão pós-parto, evidenciando associação positiva entre desrespeito e abuso durante o parto e depressão pós-parto.

A prevalência de depressão pós-parto identificada neste estudo (16,6%) é semelhante à média mundial (17%) ^4^ ^,^ ^20^, porém é inferior à observada no Brasil (26%) ^5^ e difere dos resultados encontrados em algumas regiões e cidades brasileiras. Dados de 3.065 mulheres em Pelotas, em 2015, mostraram prevalência variando entre 5,7% (depressão pós-parto moderada) e 9,4% (depressão pós-parto acentuada/grave) ^6^. Em Salvador, Bahia, em 2017, 19,8% das mulheres foram identificadas com suspeita de depressão pós-parto ^7^. Contudo, em São Luís, Maranhão, entre 2010 e 2012, foi observada prevalência de 9,1% para mães com bebês pré-termo e 11,8% para mães com bebês a termo ^21^.

A variação na prevalência pode estar relacionada a fatores econômicos e culturais. Países em desenvolvimento, como o Brasil, apresentam taxas mais elevadas de depressão pós-parto em comparação aos desenvolvidos, de forma que a média mundial pode não refletir a magnitude nacional do problema, por não considerar as características econômicas individuais dos países ^4^. No âmbito local, as diferentes prevalências identificadas também podem ser explicadas por discrepâncias de condição econômica, bem como por características culturais da população estudada em determinada cidade ou região ^22^ ^,^ ^23^. Além disso, as ferramentas de avaliação utilizadas, principalmente o ponto de corte considerado no instrumento e período no pós-parto, são fatores que podem contribuir para as diferentes prevalências da depressão pós-parto encontradas ^24^.

A maioria das pesquisas utiliza a EPDS para investigar a prevalência de depressão pós-parto ^2^ ^,^ ^4^ ^,^ ^5^ ^,^ ^22^. Contudo, outros instrumentos de autorrelato e, em menor proporção, entrevistas clínicas, também são empregados nos estudos ^24^ ^,^ ^25^, o que pode contribuir para as discrepâncias observadas entre as prevalências. Ferramentas autoaplicáveis, diferente das entrevistas clínicas, não determinam diagnósticos, apenas rastreiam sintomas de depressão, o que pode favorecer a superestimação das prevalências ^24^. Além disso, por serem baseadas em relatos pessoais, as respostas dos questionários podem ser influenciadas por crenças e normas culturais da entrevistada ^1^ ^,^ ^25^.

O ponto de corte considerado para ferramenta de avaliação também pode justificar as diferentes prevalências entre os estudos que utilizaram os mesmos instrumentos. Na EPDS, por exemplo, pontuações de 10 a 30 indicam mulheres com possível risco de depressão pós-parto ^17^, sendo que os pontos de corte mais frequentemente utilizados são iguais ou maiores que 10 e 13 pontos ^26^. Os escores de corte mais baixos apresentam maior sensibilidade, sendo empregados para encontrar o maior número de participantes que apresentem os sintomas. Por outro lado, os valores mais altos, como o adotado por esta investigação, apresentam maior especificidade, permitindo a identificação daquelas com níveis elevados da sintomatologia ^2^ ^,^ ^26^. Desse modo, as pesquisas que adotam pontos de corte mais altos, como a atual, podem apresentar prevalências menores.

É relevante, ainda, destacar que o período de tempo após o parto em que o instrumento é aplicado também influencia nos resultados. Na literatura, é possível observar que a avaliação de depressão pós-parto ocorre em períodos variados, que compreendem desde as primeiras 24 horas até um ano após o parto ^23^ ^,^ ^25^, sendo que a avaliação no terceiro mês, como neste estudo, é a mais comum ^2^ ^,^ ^25^. A prevalência de depressão pós-parto apresenta tendência crescente conforme o tempo de avaliação se aproxima do primeiro ano de pós-parto ^24^. Essas variações no período de avaliação podem ser explicadas pela falta de consenso entre manuais de diagnóstico, bem como pela identificação de pesquisas que observaram sintomatologia depressiva mesmo após o período estipulado por esses documentos ^2^.

A prevalência de pelo menos uma forma de desrespeito e abuso durante o parto observada nesta pesquisa (97,4%) é notavelmente elevada. Essa prevalência foi semelhante à encontrada no Peru ^27^ e no Nepal (100%) ^28^. No entanto, há discrepância dessa prevalência com os dados obtidos em muitos estudos internacionais e nacionais. Pesquisas realizadas na Espanha (38,3%) ^29^ e na América Latina (43%) ^30^ apresentaram prevalências significativamente inferiores à obtida nesta pesquisa. Achados semelhantes também foram encontrados no Brasil. Uma coorte em Ribeirão Preto, São Paulo, entre 2016 e 2017, constatou que 66,2% das mulheres foram expostas a abusos, desrespeitos e maus-tratos no parto ^31^. Outro estudo, realizado entre 2018 e 2019, com 698 puérperas em 21 maternidades de 14 cidades do Piauí, evidenciou que 19,8% das mulheres foram vítimas de algum tipo de desrespeito e abuso durante o parto ^32^.

Todos os estudos citados, incluindo a pesquisa atual, utilizaram ferramentas diferentes para avaliar a ocorrência de desrespeito e abuso durante o parto. A diversidade de formulários empregados limita as comparações e provavelmente contribui para variações na prevalência ^11^ ^,^ ^31^. A utilização de diferentes questionários pode ser atribuída à ausência de um instrumento padronizado mundialmente que investigue desrespeito e abuso durante o parto. Além disso, a falta de consenso em relação à terminologia e aos critérios de definição também pode comprometer a avaliação da prevalência ^11^. Desse modo, a tipologia e a avaliação adequada de desrespeito e abuso durante o parto ainda são desafios.

Em relação aos tipos de desrespeito e abuso durante o parto, os resultados desta pesquisa demonstraram que as condições do sistema de saúde e restrições (falta de políticas e recursos) foram o tipo de desrespeito e abuso durante o parto predominante, sendo relatado por praticamente todas as entrevistadas (94,7%). Esse resultado é diferente do observado em outros estudos, em que predominou o abuso verbal ^6^ ^,^ ^33^. O predomínio do abuso verbal também foi verificado no México (39,4%) ^34^ e em Pelotas (10%) ^33^. Muitas vezes, essa diferença pode ser atribuída ao fato de que as pesquisas tipicamente avaliam apenas os tipos de desrespeito e abuso durante o parto relacionados às interações dos profissionais de saúde com a mulher, negligenciando as condições do sistema de saúde e restrições ^6^ ^,^ ^33^ ^,^ ^34^ ^,^ ^35^.

Outra possível explicação para a elevada prevalência sobre as condições e restrições do local do parto pode estar relacionada ao fato de a pesquisa atual ter sido realizada com mulheres que receberam assistência durante o trabalho de parto e parto em uma maternidade pública do Nordeste. As maternidades públicas e mistas brasileiras apresentam maiores problemas de falta de equipamento e medicamentos e infraestrutura deficitária, sendo a Região Nordeste uma das mais afetadas ^36^. No Piauí, estado vizinho ao da realização da atual pesquisa, um estudo transversal realizado entre 2018 e 2019 verificou que as maternidades públicas e do interior apresentaram os maiores problemas com ausência de pediatras (69,6% públicas; 65% interior), anestesistas (56,5% públicas; 50% interior) e com a falta de medicamentos como sulfato de magnésio, ocitocina, imunoglobulina D e surfactante. Também foi observado que três estabelecimentos públicos e do interior sequer possuíam sala de parto cirúrgico e curetagem ^37^. Sabe-se que as condições do sistema de saúde e restrições podem interferir na prestação de cuidados e, consequentemente, na ocorrência de desrespeito e abuso durante o parto ^10^ ^,^ ^12^ ^,^ ^13^. Um inquérito nacional realizado no Brasil demonstrou que a prevalência de desrespeito e abuso durante o parto foi maior no setor público de saúde (36,2%) quando comparado com setor privado (28,7%) ^19^.

Relações inadequadas entre mulheres e profissionais de saúde e o não cumprimento de normas profissionais recomendadas também foram frequentemente observados neste estudo. As relações inadequadas manifestaram-se, principalmente, pela falta de apoio e perda de autonomia, enquanto o não cumprimento de normas profissionais recomendadas ocorreu, especialmente, pela falta de consentimento informado/confidencialidade e negligência/abandono. Resultados semelhantes foram encontrados em outras pesquisas ^28^ ^,^ ^32^. No Piauí, o descumprimento de normas profissionais representou 11,6% dos tipos de desrespeito e abuso durante o parto ^32^. Em Ribeirão Preto entre 2016 e 2017, a maioria das mulheres relatou que não pôde comer ou beber (30,5%) e foi impedida de ficar com acompanhante de sua escolha no momento do parto (25,5%) ^30^. Por sua vez, em Catanduva, São Paulo, em 2014, observou-se que 27,3% das mulheres foram submetidas a procedimentos obstétricos sem autorização, enquanto 16,3% relataram falhas dos profissionais no esclarecimento de dúvidas sobre o parto ^38^.

É consenso que o acesso à informação em saúde é um direito fundamental dos usuários e deveria ser mediado pelos profissionais de saúde. Essas informações podem facilitar a compreensão das mulheres sobre os cuidados empregados e, consequentemente, aumentar a participação das parturientes na tomada de decisões que envolvam sua saúde ^11^ ^,^ ^13^ ^,^ ^39^. Sabe-se, no entanto, que o direito de escolha, bem como a não submissão a procedimentos sem consentimento, ainda são reivindicações recentes e, portanto, não são tipicamente reconhecidas por profissionais de saúde, ou mesmo pelas mulheres ^40^.

Nesta pesquisa, o abuso verbal durante o parto foi relatado por 16,3% das mulheres. A ocorrência desse tipo de desrespeito e abuso durante o parto também foi evidenciada em outros estudos brasileiros ^19^ ^,^ ^39^. Uma pesquisa nacional realizada com 2.365 mulheres de 25 estados identificou que as parturientes foram submetidas a xingamentos (7%), humilhações (7%) e gritos (9%) durante atendimentos ^41^. Outro estudo, realizado entre 2013 e 2014 com 3.765 mulheres em 11 maternidades vinculadas à Rede Cegonha no Estado do Ceará, demonstrou que, no parto, as mulheres receberam apelidos (28,3%), ordens para parar de gritar (24,6%), ameaças (8,2%), críticas (6,9%), ironias (5,5%) e piadas (3,4%) ^39^. Frases sobre a sexualidade da mulher, tais como “*na hora de fazer foi bom né*... *agora aguenta!*”, “*quem entrou agora vai ter que sair!*” e “*não chora não, que ano que vem você está aqui de novo*” são abusos verbais comumente expressos pelos profissionais de saúde ^41^. Muitas mulheres não demonstram resistência aos abusos verbais por receio de os cuidados à saúde serem negados ^28^. Outras, por sua vez, sofrem em silêncio diante dos abusos, por não conseguirem perceber quando são submetidas à violência, o que pode estar relacionado à falta de conhecimento sobre os tipos de desrespeito e abuso durante o parto ^29^ ^,^ ^39^.

Nesta investigação, o abuso físico foi mencionado por 2,1% das participantes. Esse resultado é consistente com o encontrado em outros estudos brasileiros ^32^ ^,^ ^33^. Entretanto, é significativamente inferior aos achados de outros países, como Etiópia ^42^ e Nigéria ^43^, onde o abuso físico, com prevalência de 100% e 35,7%, respectivamente, foi o tipo de desrespeito e abuso durante o parto mais comum. Algumas mulheres e profissionais de saúde consideram a prática de abusos físicos como uma medida para garantir bons resultados e evitar complicações para mãe e bebê durante o parto. Além disso, é considerada necessária diante de parturientes não cooperativas e desobedientes ^28^ ^,^ ^29^. Essa visão poderia explicar a ocorrência do abuso físico evidenciado nas pesquisas, mesmo após a garantia do direito mundial às mulheres ao atendimento digno e respeitoso durante o parto.

A discriminação durante o parto, na pesquisa atual, foi manifestada em decorrência de idade, raça e/ou religião. Diversos estudos nacionais e internacionais também relataram a ocorrência de discriminação no parto relacionado à idade da mulher ^32^ ^,^ ^43^. As parturientes mais jovens, especialmente as adolescentes, são as mais afetadas pelos desrespeito e abuso durante o parto praticados por profissionais de saúde, que, por meio de uma assistência desrespeitosa e abusiva, expressam desaprovação e punem as mulheres pelo início da atividade sexual precoce, assim como as culpam pela gestação ^10^ ^,^ ^28^. A discriminação manifestada pela raça/cor da pele da mulher também é relatada com frequência nas pesquisas, sendo negras e pardas as principais vítimas ^44^. Nos Estados Unidos, entre 2010 e 2016, dados de 2.138 participantes evidenciaram que as mulheres com cor da pele branca eram menos propensas a relatar ocorrência de desrespeito e abuso durante o parto, enquanto negras e aquelas com companheiros negros foram submetidas com mais frequência à assistência desrespeitosa e abusiva ^45^. No Brasil, estudos nacionais de base hospitalar demonstraram que mulheres pretas e pardas foram as que tiveram menos chance de ter acompanhante e privacidade durante o parto ^44^ ^,^ ^46^.

Os resultados da análise multivariada não demonstraram associação significativa entre a ocorrência de pelo menos 1 tipo de desrespeito e abuso durante o parto e os sintomas de depressão pós-parto. Ao contrário do que se poderia esperar, a análise bivariada mostrou que sofrer uma forma de desrespeito e abuso durante o parto apresentou-se como fator de proteção para depressão pós-parto, cujo efeito pode ter ocorrido por falta de controle dos fatores de confundimento. Esse achado difere dos resultados encontrados em todos os estudos nacionais e internacionais que investigaram essa relação e que, por sua vez, evidenciaram associação significativa entre pelo menos um tipo de desrespeito e abuso durante o parto e depressão pós-parto ^6^ ^,^ ^7^ ^,^ ^8^ ^,^ ^9^ ^,^ ^10^ ^,^ ^14^ ^,^ ^19^ ^,^ ^47^ ^,^ ^48^. É possível que o fato de praticamente todas as entrevistas da pesquisa atual terem relatado desrespeito e abuso durante o parto do tipo “condições do sistema de saúde e restrições”, tornando isso praticamente uma característica inerente da população, tenha influenciado na associação. Portanto, é importante ter cautela ao interpretar o resultado deste estudo.

Uma revisão de escopo, realizada em 2022, demonstrou que mulheres submetidas a desrespeito e abuso durante o parto apresentam maior probabilidade de apresentar sintomas de depressão pós-parto ^9^. Um estudo multinacional realizado pela Organização Mundial da Saúde (OMS) em quatro países (Gana, Guiné, Myanmar e Nigéria), entre 2016 e 2017, mostrou que as mulheres que sofreram abuso físico ou verbal no parto tiveram aproximadamente duas vezes mais chances de terem depressão pós-parto moderada a grave ^10^. Corroborando com esse dado, uma investigação conduzida na Espanha, em 2019, evidenciou que sofrer violência psicoafetiva ou verbal aumenta a probabilidade de depressão pós-parto ^47^. Essa associação também foi identificada em estudos brasileiros realizados no Distrito Federal ^14^ e em Pelotas ^6^, que demonstraram que o desrespeito e abuso durante o parto aumenta entre 1,6 e 7 vezes as chances de desenvolver depressão pós-parto.

Por outro lado, os achados desta pesquisa demonstraram que a exposição a 2 ou mais formas de desrespeito e abuso durante o parto aumenta em aproximadamente três vezes o risco de depressão pós-parto. A literatura revela que o número de experiências de desrespeito e abuso durante o parto ao qual a mulher é submetida está diretamente relacionado à chance de desenvolver depressão pós-parto ^6^ ^,^ ^10^. Um estudo realizado na Rússia ^48^, entre 2020 e 2021, com 2.256 mulheres que deram à luz antes e durante a pandemia de COVID-19 evidenciou que a sintomatologia de depressão pós-parto foi proporcionalmente maior entre aquelas que sofreram mais tipos de desrespeito e abuso durante o parto. Outro estudo, realizado em Pelotas ^6^, revelou que as experiências de 3 ou mais tipos de desrespeito e abuso durante o parto aumentaram em três e quatro vezes, respectivamente, a probabilidade de depressão pós-parto, em comparação com mulheres que não sofreram com assistência desrespeitosa e abusiva.

Algumas limitações deste estudo devem ser consideradas. Primeiro, por ser uma pesquisa com delineamento transversal, não é possível estabelecer relação de causalidade entre desrespeito e abuso durante o parto e depressão pós-parto. Depois, a ocorrência de desrespeito e abuso durante o parto e da sintomatologia de depressão pós-parto foram baseadas na percepção das entrevistadas, não sendo avaliada diretamente pela pesquisadora. Por fim, a coleta de dados ocorreu no domicílio das participantes e, apesar de sempre ter sido solicitada uma sala reservada, muitas vezes outros moradores estavam presentes na residência, o que pode ter contribuído para subestimação da prevalência de depressão pós-parto.

Apesar dessas limitações, deve-se destacar que este é o primeiro estudo que avaliou a relação entre desrespeito e abuso durante o parto e depressão pós-parto na Região Nordeste do Brasil. Além disso, empregou-se ferramenta de rastreio validada e amplamente utilizada para avaliar sintomas de depressão pós-parto. Outro ponto que deve ser mencionado é a coleta de dados três meses após o parto. Esse período, ao contrário do que ocorre com a entrevista logo após o parto, permite que elas consigam ter tempo para processar a ocorrência de assistência desrespeitosa e abusiva ^35^. Dessa forma, os resultados encontrados nesta pesquisa contribuem para reduzir a lacuna científica relacionada ao tema, além de poder auxiliar na elaboração de estratégias institucionais locais para melhorar a assistência durante o parto, garantindo atendimento digno e respeitoso às mulheres. Ao oferecer informações sobre a prevalência de depressão pós-parto, os dados poderão ressaltar a importância do rastreio desse transtorno nas consultas de pós-parto, a fim de oportunizar tratamento para as mulheres e evitar as repercussões da doença para a mulher e seus filhos.

## Considerações finais

Os dados deste estudo revelam que a experiência de dois ou mais tipos de desrespeito e abuso durante o parto aumenta em aproximadamente três vezes o risco de depressão pós-parto. Desse modo, garantir atendimento digno e respeitoso às mulheres pode reduzir os riscos da sintomatologia de depressão pós-parto. Estratégias institucionais locais, como aumentar o investimento financeiro em infraestrutura e ofertar qualificação profissional relacionada à humanização do parto podem ser opções para melhorar a assistência às mulheres.

## References

[1] World Health Organization (2017). Depression and other common mental disorders: global health
estimates.

[2] Moraes GPA, Lorenzo L, Pontes GAR, Montenegro MC, Cantilino A (2017). Screening and diagnosing postpartum depression when and
how?. Trends Psychiatry Psychother.

[3] Gastaldon C, Solmi M, Correll CU, Barbui C, Schoretsanitis G (2022). Risk factors of postpartum depression and depressive symptoms
umbrella review of current evidence from systematic reviews and
meta-analysis of observational studies. Br J Psychiatry.

[4] Wang ZY, Liu J, Shuai H, Cai Z, Fu X, Liu Y (2021). Mapping global prevalence of depression among postpartum
women. Trans Psychiatry.

[5] Theme-Filha MM, Ayers S, Gama SNG, Leal MC (2016). Factors associated with postpartum depressive symptomatology in
Brazil the Birth in Brazil National Research Study,
2011/2012. J Affect Disord.

[6] Silveira MF, Mesenburg MA, Bertoldi AD, Mola CL, Bassani DG, Domingues MR (2019). The association between disrespect and abuse of women during
childbirth and postpartum depression findings from the 2015 Pelotas birth
cohort study. J Affect Disord.

[7] Araújo IS, Aquino KS, Fagundes LKA, Santos VC (2019). Postpartum depression epidemiological clinical profile of
patients attended in a reference public maternity in
Salvador-BA. Rev Bras Ginecol Obstet.

[8] Slomian J, Honvo G, Emonts P, Reginster JY, Bruyère O (2019). Consequences of maternal postpartum depression a systematic
review of maternal and infant outcomes. Womens Health.

[9] Conceição HN, Gonçalves CFG, Mascarenhas MDM, Rodrigues MTP, Madeiro AP (2023). Desrespeito e abuso durante o parto e depressão pós-parto uma
revisão de escopo. Cad Saúde Pública.

[10] Guure C, Aviisah PA, Adu-Bonsaffoh K, Mehrtash H, Aderoba AK, Irinyenikan TA (2023). Mistreatment of women during childbirth and postpartum depression
secondary analysis of WHO community survey across four
countries. BMJ Global Health.

[11] Leite TH, Marques ES, Esteves-Pereira AP, Nucci MF, Portella Y, Leal MC (2022). Desrespeitos e abusos, maus tratos e violência obstétrica um
desafio para a epidemiologia e a saúde pública no Brasil. Ciênc Saúde Colet.

[12] Bohren MA, Vogel JP, Hunter EC, Lutsiv O, Makh SK, Souza JP (2015). The mistreatment of women during childbirth in health facilities
globally a mixed-methods systematic review. PLoS Med.

[13] Mannava P, Durrant K, Fisher J, Chersich M, Luchters S (2015). Attitudes and behaviours of maternal health care providers in
interactions with clients a systematic review. Global Health.

[14] Souza KJ, Rattner D, Gubert M (2017). Institutional violence and quality of service in obstetrics are
associated with postpartum depression. Rev Saúde Pública.

[15] Lamy ZC, Gonçalves LLM, Carvalho RHSBF, Alves MTSSB, Koser ME, Martins MS (2021). Atenção ao parto e nascimento em maternidades do Norte e Nordeste
brasileiros percepção de avaliadores da Rede Cegonha. Ciênc Saúde Colet.

[16] Departamento de Informática do SUS Sistema de Informações sobre Nascidos Vivos - SINASC..

[17] Cox JL, Holden JM, Sagovsky R (1987). Detection of postnatal depression Development of the 10-item
Edinburgh Postnatal Depression Scale. Br J Psychiatry.

[18] Santos IS, Matijasevich A, Tavares BF, Barros AJD, Botelho IP, Lapolli C (2007). Validation of the Edinburgh Postnatal Depression Scale (EPDS) in
a sample of mothers from the 2004 Pelotas Birth Cohort Study. Cad Saúde Pública.

[19] Leite TH, Pereira APE, Leal MC, Silva AAM (2020). Disrespect and abuse towards women during childbirth and
postpartum depression findings from Birth in Brazil Study. J Affect Disord.

[20] Hanh-Holbrook J, Cornwell-Hinrichs T, Anaya I (2018). Economic and health predictors of national postpartum depression
prevalence a systematic review, meta-analysis, and meta-regression of 291
studies from 56 countries. Front Psychiatry.

[21] Eduardo JAFP, Figueiredo FP, Rezende MG, Roza DL, Freitas SF, Batista RFL (2022). Preterm birth and postpartum depression within 6 months after
childbirth in a Brazilian cohort. Arch Womens Ment Health.

[22] Corrêa H, Couto TC, Santos W, Romano-Silva MA, Santos LMP (2016). Postpartum depression symptoms among Amazonian and Northeast
Brazilian women. J Affect Disord.

[23] Hartmann JM, Mendoza-Sassi RA, Cesar JA (2017). Depressão entre puérperas prevalência e fatores
associados. Cad Saúde Pública.

[24] Míguez MC, Vázquez MB (2023). Prevalence of postpartum major depression and depressive symptoms
in Spanish women a longitudinal study up to 1 year
postpartum. Midwifery.

[25] Shorey S, Chee CYI, Ng ED, Chan YH, Tam WWS, Chong YS (2018). Prevalence and incidence of postpartum depression among healthy
mothers a systematic review and meta-analysis. J Psychiatr Res.

[26] Levis B, Negeri Z, Sun Y, Benedetti A, Thombs BD (2020). Accuracy of the Edinburgh Postnatal Depression Scale (EPDS) for
screening to detect major depression among pregnant and postpartum women
systematic review and meta-analysis of individual participant
data. BMJ.

[27] Montesinos-Segura R, Urrunaga-Pastor D, Mendoza-Chuctaya G, Taype-Rondan A, Helguero-Santin LM, Martinez-Ninanqui FW (2018). Disrespect and abuse during childbirth in fourteen hospitals in
nine cities of Peru. Int J Gynecol Obstet.

[28] Ghimire NP, Joshi SK, Dahal P, Swahnberg K (2021). Women's experience of disrespect and abuse during institutional
delivery in Biratnagar, Nepal. Int J Environ Res Public Health.

[29] Mena-Tudela D, Roman P, González-Chordá VM, Rodriguéz-Arrastia M, Gutierréz-Cascajares L, Ropero-Padilla C (2023). Experiences with obstetric violence among healthcare
professionals and students in Spain a constructivist grounded theory
study. Women Birth.

[30] Tobasía Hege C, Pinart M, Madeira S, Guedes A, Reveiz L, Valdez-Santiago R (2019). Irrespeto y maltrato durante el parto y el aborto en la América
Latina revisión sistemática y metaanálisis. Rev Panam Salud Pública.

[31] Dornelas ACVR, Rodrigues LS, Penteado MP, Batista RFL, Bettiol H, Cavalli RC (2022). Abuse, disrespect and mistreatment during childbirth care
contribution of the Ribeirão Preto cohorts, Brazil. Ciênc Saúde Colet.

[32] Madeiro AP, Rufino AC, Acaqui RF, Barbosa CM, Martins VMML, Sousa AMC (2022). Disrespect and abuse during childbirth in maternity hospitals in
Piauí, Brazil a cross-sectional study. Int J Gynecol Obstet.

[33] Mesenburg MA, Victora CG, Serruya SJ, Ponce de León R, Damaso AH, Domingues MR (2018). Disrespect and abuse of women during the process of childbirth in
the 2015 Pelotas birth cohort.. Reprod Health.

[34] Monge AB, Elorriaga MF, Verástegui OP, Santiago RV, Nolasco MAM, Alvarez IY (2022). Disrespect and abuse in obstetric care in Mexico an observational
study of deliveries in four hospitals. Matern Child Health J.

[35] Sando D, Abuya A, Asefa A, Banks KP, Freedman LP, Kujawski S (2017). Methods used in prevalence studies of disrespect and abuse during
facility-based childbirth lessons learned. Reprod Health.

[36] Bittencourt SDA, Reis LGC, Ramos MM, Rattner D, Rodrigues PL, Neves DCO (2014). Estrutura das maternidades aspectos relevantes para a qualidade
da atenção ao parto e nascimento. Cad Saúde Pública.

[37] Madeiro A, Rufino AC, Nunes MDS, Martins VMML, Barbosa CM, Sousa AMC (2022). Análise da adequação estrutural das maternidades do Piauí,
Brasil, 2018-2019. Rev Bras Saúde Mater Infant.

[38] Biscegli TS, Grio JM, Melles LC, Ribeiro SRMI, Gonsaga RAT (2015). Violência obstétrica perfil assistencial de uma maternidade
escola do interior do estado de São Paulo. CuidArte Enferm.

[39] Rodrigues FAC, Lira SVG, Magalhães PH, Freitas ALV, Mitros VMS, Almeida PC (2017). Violência obstétrica no processo de parturição em maternidades
vinculadas à Rede Cegonha. Reprod Clim.

[40] Lansky S, Souza KV, Peixoto ERM, Oliveira BJ, Diniz CSG, Vieira NF (2019). Violência obstétrica influência da Exposição Sentidos do Nascer
na vivência das gestantes. Ciênc Saúde Colet.

[41] Venturi G, Godinho T (2013). Mulheres brasileiras e gênero nos espaços público e privado: uma década
de mudanças na opinião pública..

[42] Adinew YM, Hall H, Marshall A, Kelly J (2021). Disrespect and abuse during facility-based childbirth in central
Ethiopia. Glob Health Action.

[43] Okafor II, Ugwu EO, Obi SN (2014). Disrespect and abuse during facility-based childbirth in a
low-income country. Int J Gynecol Obstet.

[44] Diniz CSG, d'Orsi E, Domingues RMSM, Torres JA, Dias MAB, Schneck CA (2014). Implementação da presença de acompanhantes durante a internação
para o parto: dados da pesquisa nacional Nascer no Brasil.. Cad Saúde Pública.

[45] Vedam S, Stoll K, Taiwo TK, Rubashkin N, Cheyney M, Strauss N (2019). The Giving Voice to Mothers study inequity and mistreatment
during pregnancy and childbirth in the United States. Reprod Health.

[46] d'Orsi E, Brüggemann OM, Diniz CSG, Aguiar JM, Gusman CR, Torres JA (2014). Desigualdades sociais e satisfação das mulheres com o atendimento
ao parto no Brasil: estudo nacional de base hospitalar.. Cad Saúde Pública.

[47] Martinez-Vázquez S, Hernández-Martínez A, Rodríguez-Almagro J, Delgado-Rodríguez M, Martínez-Galiano JM (2022). Relationship between perceived obstetric violence and the risk of
postpartum depression an observational study. Midwifery.

[48] Yakupova V, Suarez A, Karchenko A (2021). Birth experience, postpartum PTSD and depression before and
during the pandemic of COVID-19 in Russia. Int J Environ Res Public Health.

